# Optimizing Hip Abductor Strengthening for Lower Extremity Rehabilitation: A Narrative Review on the Role of Monster Walk and Lateral Band Walk

**DOI:** 10.3390/jfmk10030294

**Published:** 2025-07-30

**Authors:** Ángel González-de-la-Flor

**Affiliations:** Department of Physiotherapy, Faculty of Medicine, Health and Sport, Universidad Europea de Madrid, Villaviciosa de Odón, 28670 Madrid, Spain; angel.gonzalez@universidadeuropea.es

**Keywords:** gluteus medius, strengthening, lateral walk, side-stepping

## Abstract

**Introduction:** Hip abductor strength is essential for pelvic stability, lower limb alignment, and injury prevention. Weaknesses of the gluteus medius and minimus contribute to various musculoskeletal conditions. Lateral band walks and monster walks are elastic resistance exercises commonly used to target the hip abductors and external rotators in functional, weight-bearing tasks. Therefore, the aim was to summarize the current evidence on the biomechanics, muscle activation, and clinical applications of lateral and monster band walks. **Methods:** This narrative review was conducted following the SANRA guideline. A comprehensive literature search was performed across PubMed, Scopus, Web of Science, and SPORTDiscus up to April 2025. Studies on the biomechanics, electromyography, and clinical applications of lateral band walks and monster walks were included, alongside relevant evidence on hip abductor strengthening. **Results:** A total of 13 studies were included in the review, of which 4 specifically investigated lateral band walk and/or monster walk exercises. Lateral and monster walks elicit moderate to high activation of the gluteus medius and maximus, especially when performed with the band at the ankles or forefeet and in a semi-squat posture. This technique minimizes compensation from the tensor fasciae latae and promotes selective gluteal recruitment. Proper execution requires control of the trunk and pelvis, optimal squat depth, and consistent band tension. Anatomical factors (e.g., femoral torsion), sex differences, and postural variations may influence movement quality and necessitate tailored instruction.

## 1. Introduction

The hip is a ball-and-socket joint formed by the femoral head and acetabulum, reinforced by a fibrocartilaginous labrum, strong capsular ligaments (e.g., iliofemoral, ischiofemoral), and surrounding musculature [1]. This architecture allows triplanar motion while maintaining stability. Key muscular support comes from the gluteal group. The hip abductor muscles, chiefly the gluteus medius and gluteus minimus with assistance from the TFL and upper fibers of the gluteus maximus, are situated on the lateral aspect of the hip. The gluteus medius and minimus originate from the ilium and insert on the greater trochanter, sharing the innervation of the superior gluteal nerve. Functionally, the anterior fibers of the gluteus medius and minimus also contribute to hip internal rotation, while the posterior fibers assist in external rotation, reflecting their broad attachment and fiber orientation [2].

In weight bearing, the hip abductors are essential stabilizers. During gait and a single-leg stance, they activate to maintain a level pelvis and prevent contralateral hip drop (a positive Trendelenburg sign) [3]. By generating counteracting torque to gravity, the abductors stabilize the pelvis over the stance limb. For example, the gluteus medius can produce large forces (often over twice the body weight) to support the pelvis during mid-stance. This role is critical for normal gait mechanics, as weakness leads to a characteristic Trendelenburg gait or compensatory trunk lean [3]. Beyond frontal-plane pelvis control, the hip abductors eccentrically control hip adduction and the internal rotation of the femur during dynamic activities [4]. This helps align the lower limbs during walking, running, and jumping, protecting the knee and ankle from excessive valgus or rotation. Thus, strong and well-coordinated abductors contribute to proper posture, efficient gait, and athletic movements [5].

Optimal hip abductor function is also linked to injury prevention. Weakness or poor activation of the abductors (and external rotators) have been associated with numerous lower extremity injuries, especially in females [6,7]. Two systematic reviews found that females with patellofemoral pain syndrome (PFPS) exhibit significantly weaker hip abduction, external rotation, and extension strength compared with controls [8,9]. Prospectively, reduced hip abductor strength has been shown to increase injury risk, for instance, Leetun et al. reported that collegiate athletes with weaker hip abductors were more likely to sustain lower extremity injuries over a season [10]. Conversely, targeted strengthening of these muscles can improve lower limb kinematics and potentially reduce injury incidence [10]. Given their central role in hip stability, gait, and injury prevention, the hip abductors are a major focus in rehabilitation and conditioning programs [11]. However, despite their popularity, there is variability in how they are performed, instructed, and integrated clinically.

Therefore, this narrative review aimed to (1) summarize the relevant anatomical and functional principles of the hip abductors; (2) review the evidence on gluteal muscle recruitment during key exercises; (3) provide practical guidance on execution and clinical application across rehabilitation and performance settings.

## 2. Methods

A narrative review of the literature was conducted focusing on the anatomy, biomechanics, and clinical applications of hip abductor strengthening exercises, with a particular emphasis on lateral band walks and monster walks. This clinical commentary was conducted following the SANRA (Scale for the Assessment of Narrative Review Articles) guideline [12].

A comprehensive search was performed in PubMed, Scopus, Web of Science, and Medicine Source databases from inception to April 2025. The following search terms and Boolean combinations were used:

(“hip abduction” OR “gluteus medius” OR “hip abductors”) AND (“band walk” OR “lateral band walk” OR “monster walk” OR “elastic resistance” OR “exercise therapy”) AND (“biomechanics” OR “electromyography” OR “EMG” OR “injury prevention” OR “rehabilitation”).

In addition, the reference lists of key systematic reviews and relevant clinical guidelines were screened manually to identify additional pertinent studies.

Studies were selected based on predefined inclusion and exclusion criteria.

Inclusion Criteria:

- Peer-reviewed journal articles published in English or Spanish.
- Studies investigating hip abductor strengthening exercises, specifically lateral band walks and/or monster walks.
- Articles reporting biomechanical data (kinematics, kinetics), electromyographic (EMG) findings, or clinical outcomes related to these exercises.
- Studies examining the application of hip abductor strengthening in the prevention or rehabilitation of lower extremity musculoskeletal conditions.
- Narrative reviews, systematic reviews, meta-analyses, original research (cross-sectional, cohort, or interventional studies), and clinical guidelines.

Exclusion Criteria:

- Studies not involving hip abductor exercises.
- Studies exclusively focused on surgical interventions or post-surgical protocols unrelated to hip abductor strengthening.
- Articles lacking biomechanical, EMG, or clinical data applicable to lateral band walks or monster walks.
- Case reports, conference abstracts, or non-peer-reviewed publications (e.g., opinion pieces, letters to the editor).
- Studies addressing hip strengthening in populations with neurological conditions where gait abnormalities are primarily neurological rather than biomechanical.

## 3. Results

**Figure 1:**
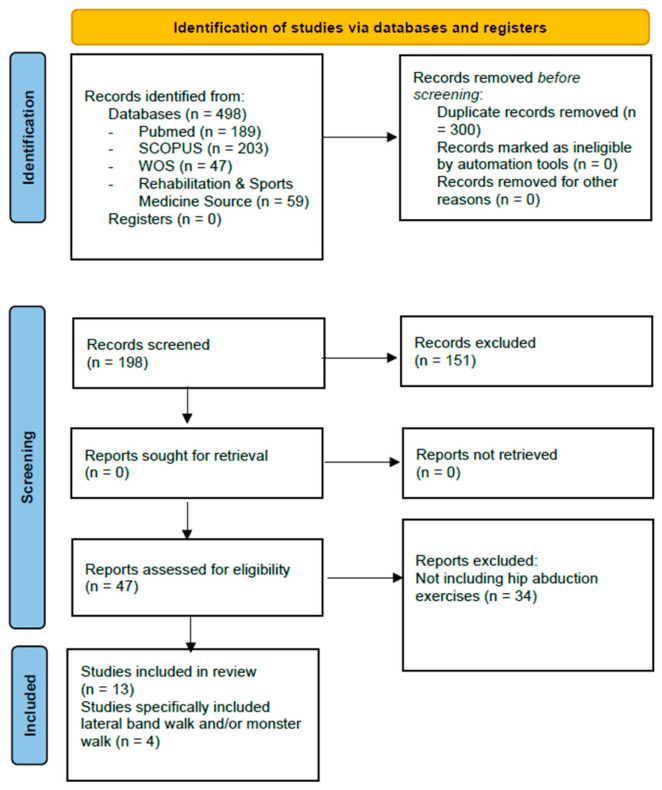
PRISMA flowchart.

The initial search yielded 498 records. Then, 198 records were screened based on the title and abstract. The full texts of 47 reports were assessed for eligibility and 13 studies were included in the review, of which 4 specifically investigated lateral band walk and/or monster walk exercises (Figure 1).

## 4. Review of Hip Abductor Strengthening Exercises

Strengthening the hip abductors can be achieved through a progression of exercises ranging from isolated, low-load movements to complex functional tasks. Electromyography (EMG) studies have quantified muscle activation in several exercises, guiding clinicians in exercise selection [13,14]. However, it is important to note that EMG amplitude alone may not directly reflect muscle force. Collings et al. [15] found poor agreement between EMG amplitude and gluteal muscle forces, suggesting that exercise selection should also consider biomechanical demands and not rely solely on EMG data.

In the early stages of rehabilitation, open-chain isolation exercises are commonly employed to selectively activate the gluteus medius and minimus while minimizing weight-bearing stress [16]. Among these, side-lying hip abduction is a widely used and effective option. Electromyographic studies have consistently demonstrated that this exercise generates high levels of gluteus medius activation, reaching approximately 80% of maximal voluntary isometric contraction (MVIC) [14]. It elicits greater muscle activation than other closed-chain or multi-joint exercises such as clamshells, lunges, and hops. Fine-wire EMG data further reveal that side-lying hip abduction effectively recruits both the posterior and middle fibers of the gluteus medius [17].

Another open-chain exercise is the clamshell, performed side lying with the knees bent, lifting the top knee (hip abduction/external rotation) often against band resistance. Clamshells tend to produce moderate gluteus medius activation (38–40% MVIC) [14], but with relatively low tensor fascia lata (TFL) activation, especially when performed with an optimal hip flexion angle [13]. A fine-wire EMG analysis by Selkowitz et al. [13] confirmed that the clamshell exercise can effectively target the gluteals while minimizing TFL contribution, making it useful for patients who have TFL dominance or IT band overuse issues. However, Moore et al. [17] also showed that clamshells elicit low activation of the anterior and middle gluteus medius segments, which may limit their effectiveness as a comprehensive strengthening exercise.

As strength and motor control improve, closed-chain and weight-bearing exercises are introduced to more directly train the abductors in functional positions. A common progression is moving to standing or quadruped exercises with elastic resistance or body weight. For example, standing hip abduction with a cable or band attached to the ankle allows resisted movement in the frontal plane. The gluteus medius activation in standing hip abduction is high (60% MVIC) while also engaging core stability. Similarly, a “fire hydrant” exercise (quadruped hip abduction/external rotation) can activate the posterior gluteus medius and gluteus maximus [14,18]. In addition, Selkowitz et al. identified the unilateral bridge as one of the top exercises for high gluteal activation with low TFL activation [13].

Dynamic balance such as single-leg squats and lateral step downs also recruit the hip abductors significantly. In a lab study, a single-leg squat led to 64% MVIC activation of the gluteus medius and a similar level in the gluteus maximus [14]. Single-leg deadlifts and lateral step-down maneuvers likewise demand strong hip abductor engagement to prevent the pelvis from dropping or the knee from valgus collapse. Collings et al. further demonstrated, using EMG-informed neuromusculoskeletal modeling, that side planks and loaded single-leg Romanian deadlifts generate some of the highest forces in both the gluteus medius and minimus [19].

Notably, Distefano et al. observed that single-limb squats and deadlifts produced far greater gluteus maximus activation (~59% MVIC) than lateral band walks or clamshells (~27–34% MVIC for glute max) [14] Other common hip abductor exercises include hip hikes (pelvic drop) from a step (to train the lateral hip muscles eccentrically and concentrically), side planks with hip abduction (an advanced move that combines core and hip abductor strength; known to elicit very high gluteus medius activity often > 75–100% MVIC in trained individuals [16]), and plyometric or agility drills that require lateral push off. A systematic review by Ebert et al. [16] synthesized these exercises into a graded rehabilitation continuum, noting that patients should progress from low-load activation (e.g., isometrics, side lying raises) to more challenging weight-bearing and plyometric exercises. In summary, a variety of exercises are available to strengthen the hip abductors. Isolated movements like side lying abduction or clamshells effectively target the gluteus medius early on, while functional exercises like band walks, single-leg squats, and step downs prepare the patient for sport-specific demands [7,14,16].

## 5. Lateral Band Walk and Monster Walk Exercises

Lateral band walks and monster walks are resistance band exercises designed to strengthen the hip abductors and external rotators in a functional stance [20,21,22]. Both involve using elastic bands around the legs to provide lateral resistance as the individual moves, thereby engaging the gluteal muscles to overcome the band tension. These exercises have gained popularity in sports injury prevention programs and rehabilitative protocols for their ability to activate the gluteus medius in standing positions that mimic real-life movements.

For a lateral band walk, an elastic band loop is typically placed around the legs (common placements are at the ankles or just above the knees). The individual assumes a semi-squat position (feet about shoulder-width apart, knees and hips bent ~30°) and steps sideways in a controlled manner. The technique involves taking a step to the side with the leading leg, followed by the trailing leg, all while maintaining tension on the band and keeping the feet apart (to not lose resistance) [20,21,22]. The monster walk is a variation where instead of purely side-to-side steps, the person moves in diagonal or forward/backward patterns. A classic monster walk involves stepping forward and outward (around 45° diagonal) with each foot in an alternating fashion, still in a semi-squat posture with band resistance. The term “monster” comes from the somewhat stiff-legged, wide-stance appearance. Monster walks can also be performed backward and diagonally to target the hip musculature through a slightly different range. Monster walks combine frontal and sagittal plane movement; the legs move apart and forward/back, requiring the hip abductors and external rotators to work together to control the band. This diagonal pattern can simulate sport-specific movement patterns (like cutting or defensive shuffling in athletics) [20,21,22].

Unlike open-chain abduction, band walks keep the feet on the ground and require the co-contraction of multiple muscle groups such as the stance leg’s abductors and external rotators and trunk muscles to maintain balance and posture. EMG analyses have shown that lateral band walking produces meaningful activation in the stance limb gluteus medius (30–50% MVIC) as well as the moving limb gluteus medius (20–30% MVIC) [20,21,22]. The gluteus maximus also contributes, particularly the upper fibers that assist hip abduction and external rotation. One study using surface EMG recorded gluteus maximus activation around 20–30% MVIC during band walks [14], which is lower than in single-leg squats. EMG comparisons have found that lateral band walks and monster walks can produce similar or even higher gluteus medius activation than some traditional exercises. For instance, one fine-wire EMG study noted that a properly performed lateral band sidestep activated the gluteus medius comparably to a unilateral bridge and more than certain clamshell variations [13].

## 6. Biomechanical Analysis

A biomechanical understanding of monster walks and lateral band walks helps optimize their execution and adaptation to individual needs. During a lateral band walk, the primary kinematic motions are hip abduction (moving leg) and slight hip adduction control (stance leg), along with knee flexion/extension and minimal trunk sway [22]. Each step involves the eccentric activation of the stance limb and the concentric abduction of the lead limb to counter lateral band resistance, with monster walks additionally challenging sagittal and rotational control [20,23].

Band placement is a key variable that influences muscle recruitment during lateral resistance exercises. Distal positions, such as the ankles or feet, significantly increase the lever arm and resistance torque, leading to greater activation of the gluteus medius and maximus in a dose-dependent manner [20,23]. For example, Cambridge et al. demonstrated that as the band was moved from above the knees to the ankles to the feet, gluteus medius EMG activity progressively increased. The monster walk often specifically uses an ankle or forefoot placement, which not only provides lateral resistance but also introduces a slight external rotation torque (especially if the band is around the forefoot, tending to pull the toes inward) [23]. Notably, placing the band around the forefoot imposes additional internal rotation torque, which selectively enhances gluteus maximus activation while minimizing the recruitment of the TFL [23]. Although TFL activation increases from knee to ankle placement, it does not further increase at the foot level, suggesting that foot placement maximizes gluteal engagement without excessive TFL compensation [20,21,22,23]. Band placement at the distal leg elicits greater gluteal muscle activation. From a clinical standpoint, band-at-knee placement may serve as an appropriate starting point for deconditioned individuals, with progressive distal placement recommended as strength improves [23]. Overall, forefoot placement yields the highest activation of the gluteus medius and maximus with relatively lower TFL involvement, making it the most effective configuration for selective gluteal loading [20,21,22,23].

Trunk and pelvic positioning modulate hip muscle recruitment during band exercises. A slight forward trunk lean increases gluteus medius and maximus activation, while excessive sway or lateral trunk lean may reduce abductor demand by mechanically offloading the stance limb [21]. The optimal technique involves maintaining frontal plane neutrality with a mild hip hinge to enhance posterior gluteal engagement and core stability.

Squat depth influences gluteal activation during band walks. Moderate hip and knee flexion (~30°) optimizes gluteus medius recruitment while minimizing TFL involvement. Excessive flexion may shift the effort toward the gluteus maximus, whereas an upright posture reduces overall gluteal engagement and promotes compensatory trunk movements. A semi-squat position offers the most biomechanically efficient strategy for targeting the lateral hip [21].

Anatomical variations such as femoral torsion influence movement quality during band walks [24,25,26]. Individuals presenting with excessive femoral anteversion often exhibit an increased internal rotation of the femur, particularly with distal band placement, requiring closer attention to cueing and potentially starting with proximal resistance. In contrast, individuals with femoral retroversion typically display a more externally rotated hip alignment, potentially facilitating the improved execution of the exercise with less compensatory movement [22].

Sex-based differences have been observed in the biomechanics of resisted side stepping. Women demonstrate greater gluteus medius and TFL activation than men, along with increased trunk flexion and hip abduction excursion—possibly compensating for lower absolute strength [20]. Although both sexes benefit similarly from distal band placement, females may fatigue more quickly or exhibit compensatory patterns, warranting individualized progression in load and volume. Given the higher prevalence of hip-related risk factors for knee pathologies in women, proper technique emphasizing gluteal over TFL recruitment is essential.

In summary, the monster walk and lateral band walk are influenced by several biomechanical factors: band placement (knees vs. ankles vs. feet) alters resistance distribution, stance limb vs. moving limb dictates which side works harder, and posture (trunk lean, squat depth) affects muscle recruitment patterns. Distal band placement and a semi-squat posture maximize gluteus medius and maximus activation while minimizing TFL involvement. Proper form (minimal pelvic sway, controlled steps) ensures the targeted muscles are effectively engaged.

## 7. Clinical Applications

In conditions such as patellofemoral pain syndrome (PFPS) and iliotibial band syndrome (ITBS), hip abductor weakness is a well-documented contributor to aberrant femoral kinematics, increased dynamic knee valgus, and excessive lateral stress [8,9,27,28,29]. Lateral and monster walks enhance frontal-plane control and reduce TFL overactivity, particularly when performed with a distal band placement and slight squat posture. These exercises often serve as a progression from early-stage activation drills to dynamic functional retraining. For patients with femoroacetabular impingement (FAI) and labral pathology, hip abductor training supports pelvic stability and may mitigate impingement-related symptoms by limiting compensatory movement patterns [30,31,32]. Lateral and monster walks can be used to improve neuromuscular control while avoiding the ranges of motion that provoke pain. In cases of gluteal tendinopathy, where compressive and tensile overload affects the gluteus medius and minimus tendons, these exercises offer a means of controlled, progressive loading in neutral hip positions [7,18,33]. Lateral band walks, when properly dosed and executed with attention to stride length and pelvic alignment, can enhance tendon capacity and reduce pain. Following anterior cruciate ligament (ACL) reconstruction, hip abductor strengthening contributes to restoring dynamic knee stability and correcting faulty movement patterns, such as medial knee collapse [34,35]. Lateral and monster walks are effective in re-establishing gluteal engagement during tasks that demand frontal and transverse plane control, especially as patients progress toward running and jumping. Finally, in chronic ankle instability (CAI), hip abductor weakness impairs postural control and contributes to recurrent sprains [36,37]. Incorporating these exercises enhances proximal stability, complements balance training, and may improve neuromuscular coordination across the kinetic chain.

Finally, an emerging area of research suggests a functional relationship between hip muscle strength and pelvic floor function. The hip abductors, among other hip muscles, contribute to lumbopelvic stability and may influence pelvic floor behavior, particularly in women with pelvic floor dysfunction or urinary symptoms. Recent studies have shown that hip abductor and external rotator weakness is associated with urinary incontinence, urgency, and sexual dysfunction [38,39]. These findings support the inclusion of hip abductor strengthening, through exercises such as lateral band walks and monster walks, as part of comprehensive pelvic floor rehabilitation strategies.

Monster walks and lateral band walks offer a versatile, evidence-informed approach to reinforcing hip control across diverse clinical populations. Their integration into rehabilitation programs supports improved movement quality, functional performance, and long-term injury risk reduction. Based on the current evidence and clinical experience, Table 1 show recommendations for using monster walks and lateral band walks.

## 8. Limitations

This narrative review has several limitations. As a non-systematic review, the search strategy, while comprehensive, did not follow a fully systematic process such as PRISMA. Although the SANRA guideline was followed, there remains a degree of selection and reporting bias inherent to narrative reviews. The available literature on lateral band walks and monster walks is relatively limited and heterogeneous, with varying study designs, small sample sizes, and differences in EMG methodology and exercise protocols. As such, generalizing the findings across all populations must be performed cautiously. While biomechanical and EMG findings provide valuable information about muscle activation, they do not directly translate to clinical outcomes; further high-quality longitudinal studies are needed to confirm the long-term effectiveness of these exercises in specific rehabilitation contexts.

## 9. Conclusions and Recommendations

The hip abductor musculature, particularly the gluteus medius and minimus, is crucial for maintaining pelvic stability, controlling lower limb alignment, and preventing injury. Weakness or the poor coordination of these muscles is implicated in numerous conditions (from PFPS and ITB syndrome to gluteal tendinopathy, FAI, ACL injuries, ankle instability, or pelvic floor dysfunction). Exercises like the lateral band walk and monster walk have emerged as effective tools to specifically strengthen the hip abductors in functional positions.

In clinical practice, monster walks and lateral band walks have shown their value across rehabilitation and performance settings. They help correct faulty movement patterns by reinforcing lateral hip stability, for example, reducing hip adduction/internal rotation in those with knee pain or improving balance in those with ankle instability. Importantly, they serve as a bridge between isolated strengthening and complex movement. A patient can progress from table exercises to band walks, and then to jumping or sport-specific drills, with the confidence that their hip abductors are now better trained to handle those demands.

## Figures and Tables

**Table 1:** Clinical guidelines for optimizing execution and progression of lateral and monster band walk exercises.

Parameter	Recommendations
**Band Placement**	Prefer distal placement (ankles or forefeet) to maximize gluteus medius and maximus activation. Initiate at knee level for beginners or in cases of significant weakness or pain, progressing distally as strength improves [20].
**Posture**	Perform in a mini-squat (20–30° knee and hip flexion), with slight forward trunk lean (hip hinge). Maintain an upright chest and engaged core to optimize gluteal activation and minimize tensor fascia latae involvement [22].
**Movement Execution**	Execute slow, controlled steps maintaining consistent band tension. Cue patients to avoid foot drag and ensure continuous resistance. Steps should be shoulder-width apart; knees aligned over feet; lead with heels while keeping toes forward.
**Stance Leg Emphasis**	Prioritize training the weaker side by emphasizing stabilizing roles during lateral walks. Generally perform the exercise in both directions to promote symmetry unless clinical indications suggest otherwise [23].
**Volume and Dosage**	Recommended frequency: 2–4 times/week. In rehabilitation, perform 3 sets of 10–15 steps per direction or 1 min sets. In performance contexts, 1–2 sets can be used during activation warm-ups. Emphasize movement quality over volume. Progress resistance or repetitions as endurance improves [40].
**Integration with Other Exercises**	Incorporate as part of a comprehensive program including sagittal and transverse plane hip strengthening, mobility exercises, and, for athletes, power and balance training. These walks pair well with agility drills to translate strength gains into functional performance [41].
**Monitoring and Progression**	Continuously assess for adverse responses. In cases of patellofemoral pain or gluteal tendinopathy, verify technique and adjust resistance or stride as needed. Progress only when the current level is acquired without pain.

## Data Availability

No new data were created or analyzed in this study.
